# Efficacy analysis of pedicle screw internal fixation of fractured vertebrae in the treatment of thoracolumbar fractures

**DOI:** 10.3892/etm.2013.914

**Published:** 2013-01-21

**Authors:** WEIJIE HUANG, TAO LUO

**Affiliations:** Department of Orthopedics, Shanghai Punan Hospital of Pudong New Area, Shanghai 200125, P.R. China

**Keywords:** vertical stress, pedicle screw, fractured vertebra

## Abstract

The present study aimed to discuss the method and effect of posterior internal fixation of thoracolumbar fractures strengthened by the vertical stress pedicle screw fixation of fractured vertebrae. Patients with single thoracolumbar fractures were examined retrospectively. Fourteen patients (group A) had been treated with vertical stress pedicle screw fixation of a fractured vertebra and sixteen patients (group B) received traditional double-plate fixation, as a control. All patients were diagnosed with fresh fractures with a complete unilateral or bilateral pedicle and no explosion of the inferior half of the vertebral body or inferior endplate. In group A, patients received conventional posterior distraction and lumbar lordosis restoration, as well as pedicle screws in the fractured vertebra in a vertical direction to relieve stress to achieve a local stress balance. All patients were followed up postoperatively for 4–18 months (average, 12.6 months). The vertical stress pedicle screw fixation assisted in the reduction of vertebrae fracture, which reduced the postoperative Cobb’s angle loss. There was a significant difference in the change of Cobb’s angle between the two groups one year after surgery (P<0.01). Conditional application of pedicle screws in a single thoracolumbar fracture enhances the stability of the internal fixation system and is conducive to the correction of kyphosis and maintenance of the corrective effects.

## Introduction

The incidence of spinal fractures accounts for 5–6% of whole-body fractures and the majority of these are thoracolumbar fractures (1). With the high-speed development of society, the number of high-energy injuries is increasing, including falling from high places, traffic accidents and bruises. Thoracolumbar spine injuries often involve the spinal column and fragment retrusion causes spinal canal stenosis or spinal cord and *cauda equina* injuries. Surgeons must determine whether the spine is able to withstand the physiological load and stress, and if not, spinal osseous stability must be rebuilt. Biomechanical studies on spinal stability and innovation of spinal fixation methods created the foundations for achieving the therapeutic goal (2). With the wide application of pedicle screw technology, posterior short-segmental fixation has become a reliable method in the treatment of thoracolumbar fractures. In thoracolumbar burst fractures, pedicle screw placement between the adjacent upper and lower vertebral bodies is normally used for vertebral reduction and fixation (dual-plane fixation). This method opens the fractured vertebra and restores the height of the vertebral bodies. However, there are risks of postoperative kyphosis and failed internal fixation as a result of intervertebral indirect reduction and fixation. Postoperative failure of internal fixation and the loss of the corrective angle have become important factors affecting treatment efficacy. Theoretical and experimental studies of short-segmental pedicle screw placement in the treatment of fractured vertebrae in thoracolumbar fractures began in 1994 when Dick *et al*(3) produced a biomechanical study with this method. Since then, few in-depth studies on this technology have been produced and clinical reports on the treatment of thoracolumbar fractures using this technology are rare. In this study, the admitted patients with thoracolumbar fractures were retrospectively studied to assess the value of applying vertical stress pedicle screws in the fractured vertebra.

## Materials and methods

### General data

From March 2008 to January 2010, a total of 30 patients with single thoracolumbar fractures, receiving vertical stress pedicle screw fixation of fractured vertebrae (group A) or traditional double-plate fixation (group B), were retrospectively reviewed. In group A, 11 men and 3 women, aged 33–59 years (average 46.4 years) were enrolled and 12 men and 4 women, aged 34–63 years (average 47.2 years) were enrolled into group B. The injured segments were T_12_ in 3 patients, L_1_ in 6 patients, L_2_ in 3 patients and L_3_ in 2 patients in group A and T_12_ in 3 patients, L_1_ in 7 patients, L_2_ in 4 patients and L_3_ in 2 patients in group B. All patients had fresh fractures with a complete unilateral or bilateral pedicle and no explosion of the inferior half of the vertebral body or inferior endplate. According to load sharing classification put forward by McCormack *et al*, a score <7 was an indication of simple posterior internal fixation (4). All the cases selected in this study had a score <7, with the exception of 2 patients in group A that had a score >7, who received posterior surgery for economic reasons. Preoperative and postoperative lateral X-ray film, pedicle computerized tomography (CT) scan and two- and three-dimensional reconstruction were used to evaluate the fractures and the postoperative stability of the internal fixation. In group A, patients received conventional posterior distraction and lumbar lordosis restoration, as well as alteration of ventral pressures using pedicle screws in the fractured vertebra in a vertical direction to relieve stress, achieving a local stress balance. In group B, the cephalad and caudal area of the fractured vertebra was fixed according to the traditional method, without applying vertical stress pedicle screws. This study was conducted in accordance with the declaration of Helsinki. This study was conducted with approval from the Ethics Committee of Shanghai Punan Hospital of Pudong New Area. Written informed consent was obtained from all participants.

### Surgical method

After receiving general anesthesia, patients were placed in the prone position with U-shaped pillows under their chests and bilateral iliac to impend their abdomens in order to reduce intraoperative bleeding. Following intraoperative X-ray fracture location with a C-arm fluoroscopic device, a posterior median incision was made with the fractured vertebra at the center to expose the vertebral plate and the articular process layers. Two pedicle screws were implanted into the upper vertebral body of the fractured vertebra by the Magerl method and the lower vertebral body by the Krag method, respectively. Two short nails or universal nails were implanted into the fractured vertebra and the pre-bent connection rod was attached. The upper and lower pedicle screws were distracted and the upper and median pedicle screws were locked to the connection rod. Then, the lower and median pedicle screws were longitudinally distracted to recover the fractured vertebral body height and the connection was locked. The dual-plane fixation group received longitudinal distraction and reduction following implantation of the pedicle screws into the upper and lower vertebral bodies of the fractured vertebra and attachment of the pre-bent connection rod. According to the location of the vertebral canal blockage, patients received total laminectomy decompression to remove the dural sac oppression and promote the bilateral intertransverse bone graft fusion if necessary.

### Postoperative treatment

The drainage tubes were kept in place for 24–48 h after surgery. Patients were encouraged to exercise their lumbar muscles in postoperative week 3 and perform out-of-bed activities with a brace in postoperative months 1–2. An X-ray and CT scan were taken regularly. The internal fixation was kept in place for one year after surgery.

### Statistical analysis

Contrast analysis was performed on Cobb’s angle changes and vertebral body height in postoperative two weeks with internal fixation. The vertebral body height was determined by the ratio of the mean of the fractured vertebral body and anterior heights of the adjacent two vertebral bodies. Cobb’s angle was determined by the angle formed by the perpendiculars of the extension lines of the upper endplate of the upper fractured vertebra and the lower endplate of the lower fractured vertebra. The changes to the preoperative and postoperative parameters were analyzed by independent samples t-test with SPSS 13.0 software package (SPSS Inc., Chicago, IL, USA) and all tests were two-sided. P<0.05 was considered to indicate a statistically significant difference.

## Results

There were no significant differences in Cobb’s angle and vertebral body height between the two groups preoperatively, which may rule out the bias of therapeutic effect caused by the varying degrees of instability of the fractured vertebra. There were also no significant differences in Cobb’s angle and vertebral body height between the two groups postoperatively. There was a significant difference in Cobb’s angle, but not in vertebral body height between the two groups one year after surgery (Table I).

**Figure 1.**
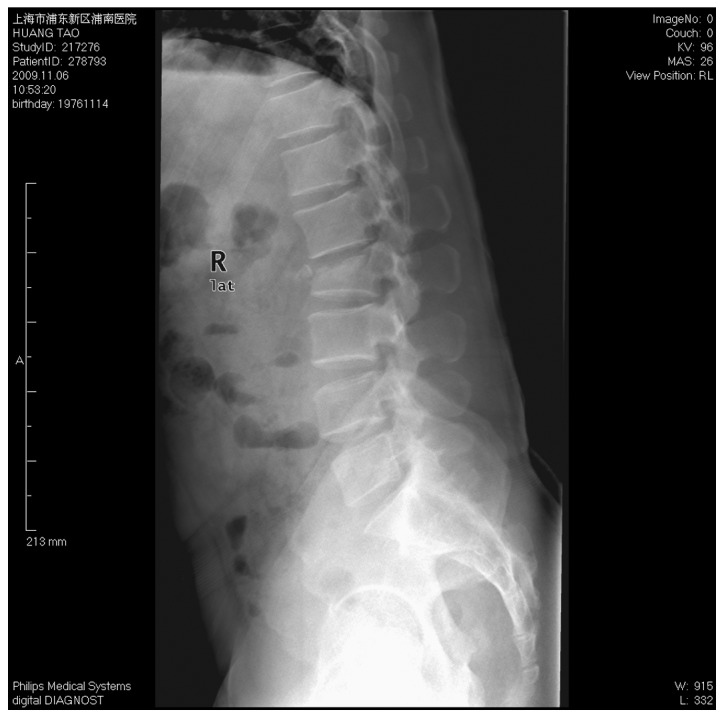
Lateral radiograph prior to triplane fixation of an L2 fracture.

**Figure 2.**
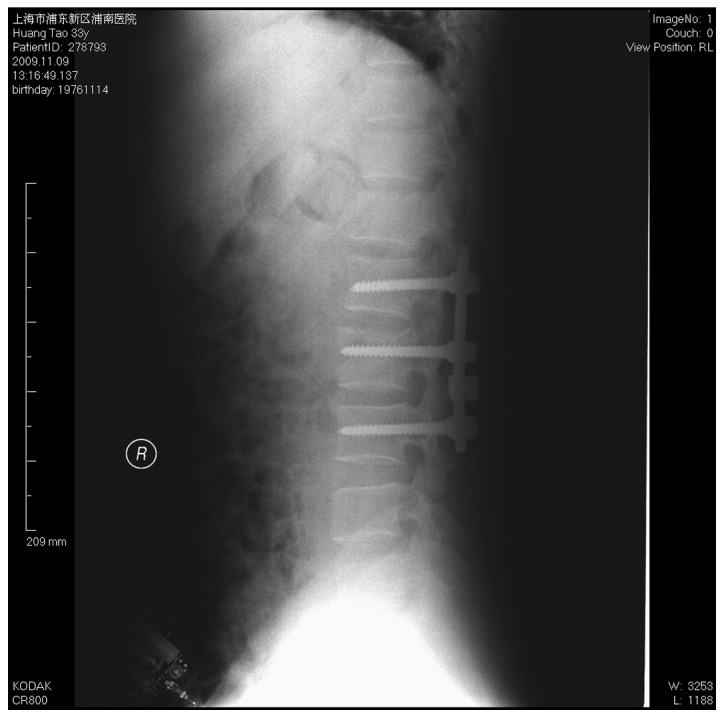
Lateral radiograph following triplane fixation of an L2 fracture.

**Figure 3.**
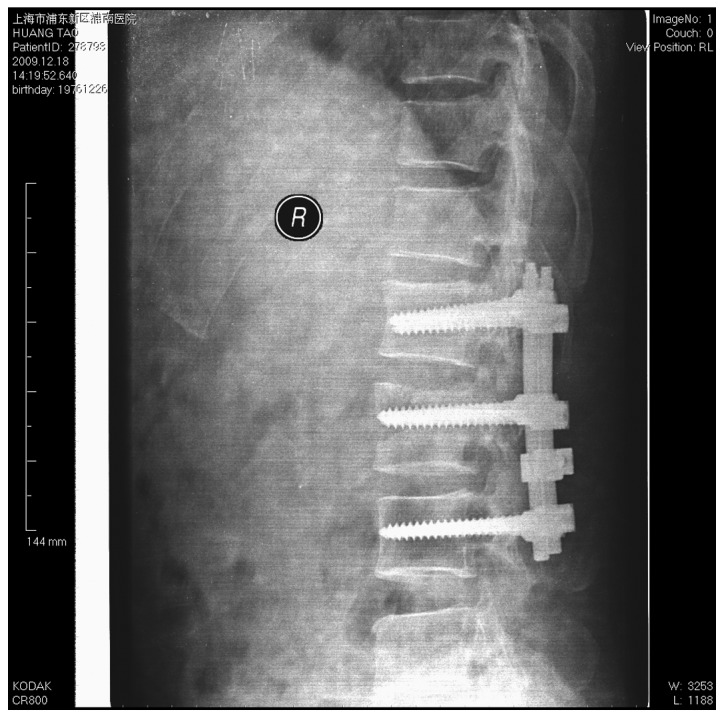
Lateral radiograph following triplane fixation of an L2 fracture for one year.

**Figure 4.**
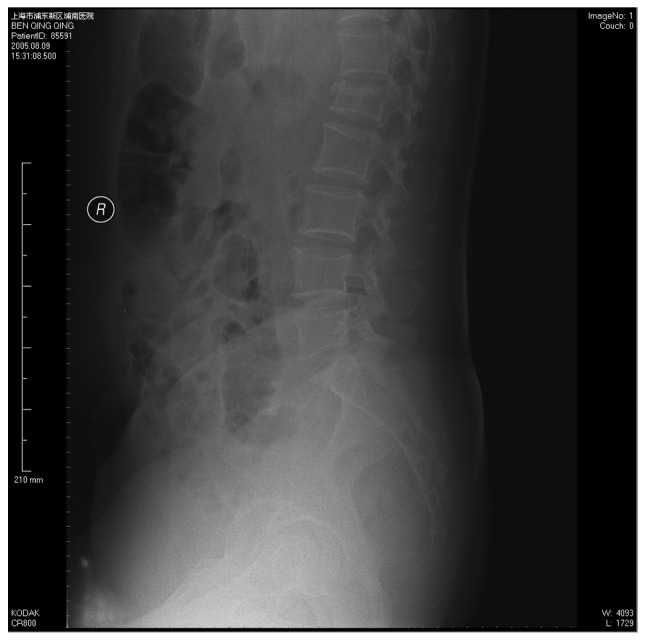
Lateral radiograph prior to dual-plane fixation of an L1 fracture.

**Figure 5.**
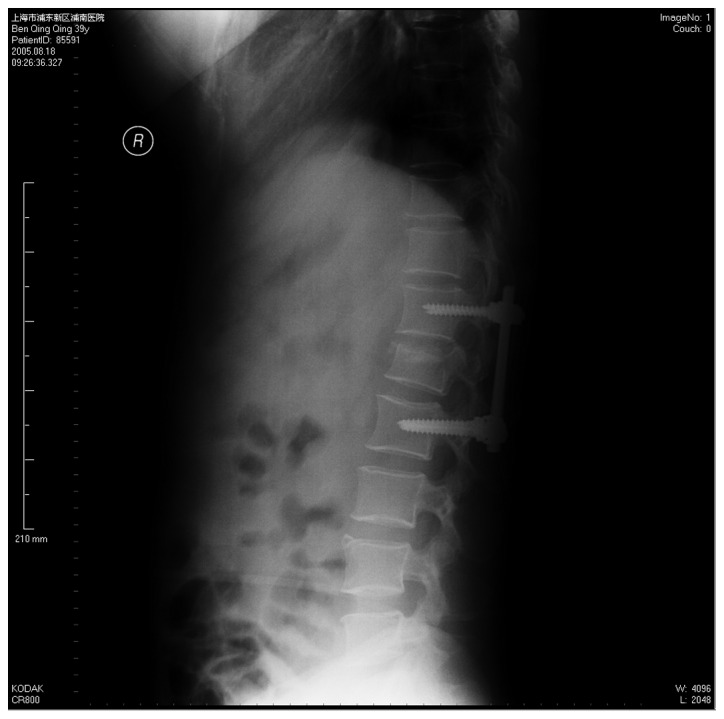
Lateral radiograph following dual-plane fixation of an L1 fracture.

**Figure 6.**
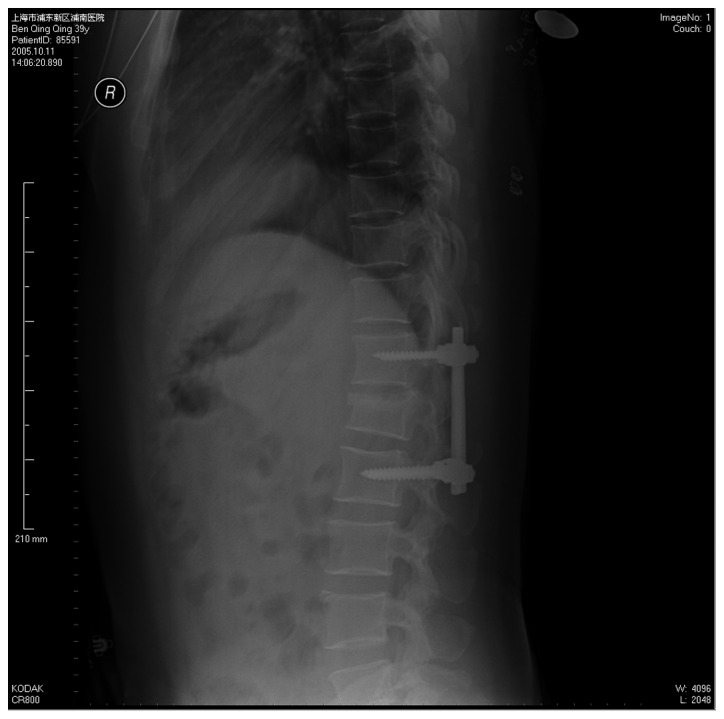
Lateral radiograph following dual-plane fixation of an L1 fracture for one year.

The results of the follow-up demonstrated whether traditional dual-plane fixation or triplane fixation with stress screw fixation through the fractured vertebra obtain a satisfactory reduction of vertebral burst fracture (Figs. 1–3), recovery of fractured vertebral height and correction of the forward bending of the spine (Fig. 3). However, the long-term follow-up revealed that the two methods effectively maintain vertebral body height and the vertical stress screw method maintained the physiological curvature of the spine (Figs. 4–6).

## Discussion

Spinal fractures cause damage to the normal structure of the spine and affect nerve function. Therefore, treatment of spinal fractures aims to recover the normal anatomical structure, remove nerve oppression and promote the recovery of nerve function. There are a number of conflicting ideas on specific treatment, including surgical or non-surgical treatment, anterior fixation or posterior fixation, long-segment or short-segment fixation and fusion or non-fusion (5–8). Spinal fractures are divided into stable and unstable fractures. The diagnostic standards of unstable fractures include: i) anterior vertebral compression >50%; ii) Cobb’s angle for kyphosis >20°; and iii) a vertebral canal blockage area larger than 50% (9,10).

Anterior surgery has the advantage of thorough decompression, which effectively corrects the kyphosis and receives good bone graft fusion, in order to establish the fused segment. However, this surgery may cause large traumas that easily damage the large blood vessels and other organs, as well as complications caused by thoracotomy and laparotomy, including intestinal adhesion and pneumothorax. It is recommended that compressed fractures and mild violence fractures receive posterior surgeries. The posterior fixation fusion through the use of pedicle screws is a simple surgery, resulting in little injury and has a rapid recovery; therefore, it is widely applied in the clinic (11).

McCormack *et al* studied various load distributions in the reconstruction of spinal fractured implants (4). It was considered that the anatomical characteristics of the fracture itself are more important than the types of implants used. In the fixation of long fractured bones, the internal fixation system and load distribution between the host bones are the basis of fracture healing and internal fixation failure. The treatment of thoracolumbar fractures should also consider the load distribution. Without reasonable load sharing, the risks of internal fixation failure and fracture healing failure significantly increase. Based on this, McCormack *et al* presented a load sharing classification of spinal fractures, which aids accurate assessment of the stability of the spine after being fractured and guides method selection for internal fixation. Additionally, this reduces the risk of internal fixation failure and improves the effects of surgery. Scoring is performed according to the vertebral body involved in the fracture, the displacement of the fracture parts and the kyphotic deformity (4). Parker *et al* considered that a score of <6 points presents a good load sharing capability and simple posterior pedicle fixation could achieve a good stability. A score >7 points presents a poor load sharing capability and in simple posterior pedicle fixation, there is a risk of internal fixation failure, including a broken nail. In these cases, the fixation should be replaced with anterior bone graft fusion or two-stage anterior surgery (12).

Whether fractured vertebrae require vertical stress screw fixation to assist and maintain the reduction remains unclear. Traditional dual-plane fixation that fixes the normal upper and lower vertebral bodies of fractured vertebrae has the following problems: distraction of the fractured vertebral height results in poor recovery and the intervertebral height increases, particularly the non-affected ones; the fixation has a parallelogram effect and a lateral instability so it often requires the addition of a transverse connection fixation; and finally the fixation has a suspension effect, whereby decreasing the distance between the upper and lower anterior vertebral bodies and increasing recession of intermediate fractured vertebrae.

The pedicle internal fixation of fractured vertebrae is technically feasible and effective in restoring vertebral body height and correcting dislocation (13,14). The screw implantation through fractured vertebrae significantly improves the stress distribution of screws, reduces screw load and provides a fulcrum for the reduction to make it coincide with the mechanical mechanism, so as to significantly improve its anti-stress ability and significantly enhance the stability of the fixation (15). In the stress screw fixation through fractured vertebrae, an appropriate amount of ventral pressure overcomes the kyphosis stress caused by fractures, which is helpful for maintaining the physiological curvature of fixed parts postoperatively to prevent the screws from loosening. This may also benefit and reduce dislocation for patients with fracture dislocation. The stress added between the screw and pedicle is a ventral pressure stress but not a pullout force. Complete pedicle fixation is enough to guarantee the stability of the vertical stress screw (16).

Dick *et al*(3) reported the comparison of biomechanical experiments of 6-screw and 4-screw fixation in a cattle lumbar model and identified that the 6-screw fixation has clear advantages. The axial load capacity increases 160%, the bending resistance capacity increases 48% and the torsional rigidity increases by 38%. The authors demonstrated that screw implantation through the fractured vertebra increases the effect of resistance to stress. Conversely, Hakalo and Wronski (17) considered the there is no basis for fractured vertebrae fixation. Following spinal fracture, the reduction of the axial ligament could rearrange the bones connected to the ligament and restore the shape of injured vertebrae; however, a ‘shell effect’ still existed in fractured vertebrae following reduction. The fractured vertebra and its upper and lower clearances did not have weight-bearing capacity and the load was mainly conducted through the internal fixation. Therefore, pedicle screw implantation through the fractured vertebra did not effectively increase the spinal axial bearing capacity and initial stability, thus it does not reduce the postoperative corrective loss and failure rate of internal fixation. Thoracolumbar fractures not accompanied with anterior and posterior longitudinal ligament fracture are given inter-segmental pedicle screw fixation and distraction. The stability is enough to meet the clinical needs and patients do not require re-fixation of the fractured vertebra. Fractured vertebrae fixation not only failed to clearly increase the stability of the fixed segment, but also increased the surgery time, as well as surgery risk and economic burden of patients. However, the *in vitro* study did not consider the role of the neuromuscular system and other stable structures on the stability of the spine. The early postoperative evaluation lacked long-term effect.

Maintenance of fractured vertebral height and spinal curvature are important in the treatment of vertebral fractures. With the distraction and reduction of the posterior pedicle screw, seriously collapsed vertebral fractures demonstrate clear recovery of vertebral body height intraoperatively under a C-arm fluoroscopic device or in an X-ray film. However, the ‘shell effect’ of fractured vertebrae still exists postoperatively in internal fixation. The compressed trabeculae in the vertebral body do not achieve complete reduction. The residual interspace is difficult to heal. Therefore, if the stability of the anterior and median column are not reestablished quickly, the posterior internal fixation takes continuous and excessive loads, increasing the risk of corrective angle loss and internal fixation failure (18,19).

In this study, we observed satisfactory postoperative recovery of vertebral body height in the triplane fixation group and dual-plane fixation group, with 92.9 and 90.9% recovery, respectively. There was also relatively good postoperative correction of spinal kyphosis, with a postoperative Cobb’s angle of 2.51 and 3.26°, respectively. There were no significant differences between the two groups. One year after surgery, the vertebral body height in the two groups was 91.4 and 89.1%, respectively, without a significant difference. However, the Cobb’s angles were 2.51 and 5.12°, respectively, with a significant difference. This indicates that triplane fixation and dual-plane fixation maintain the vertebral body height and that triplane fixation is more effective at maintaining the Cobb’s angle and preventing kyphosis of the spine.

The advantages of using pedicle screws to treat fractured vertebrae include the following: i) it provides a good three-point fixation to reduce the suspension effect of the internal fixation system; ii) it reduces the parallelogram effect to increase the stability; iii) it avoids stretching the normal intervertebral disc, which is beneficial to the recovery of the vertebral fracture form and iv) it dispenses the stress of the pedicle screw connection. Therefore, conditional application of vertical stress screw fixation of fractured vertebrae enhances the stability of the posterior short-segment internal fixation system for thoracolumbar fractures and facilitates the correction of kyphosis and maintenance of the corrective effect (20,21).

In our comparative study of the two groups, we demonstrate that the two internal fixation methods maintain vertebral body height and that vertical stress screw fixation of fractured vertebrae is more effective at maintaining spinal postoperative physiological curvature of the spine and reducing the angle loss. Conditional application of vertical stress screw fixation of fractured vertebrae enhances the stability of the posterior short-segment internal fixation system for thoracolumbar fractures and facilitates the correction of kyphosis. The limitations of this study are that it is a retrospective study with a small sample size and short follow-up period. A multicenter, large sample study with a long follow-up is required in order to achieve a more definite conclusion.

## Figures and Tables

**Table I.** Comparison of relevant parameters in the two groups.

	Group A	Group B	t-value	P-value
Preoperative Cobb’s angle (°)	9.63±2.01	9.07±1.87	0.780	0.442
Preoperative vertebral body height (%)	50.68±5.89	50.8±8.35	−0.073	0.942
Postoperative Cobb’s angle (°)	2.51±1.14	3.26±1.91	−1.263	0.217
Postoperative vertebral body height (%)	92.92±5.14	90.9±4.99	1.073	0.293
Cobb’s angle in postoperative one year (°)	2.51±1.25	5.12±1.07	−6.512	0.001^a^
Vertebral body height in postoperative one year (%)	91.43±4.99	89.1±2.74	1.552	0.132

a Significant difference between the two groups.
